# Circulating tumor cells from melanoma patients show phenotypic plasticity and metastatic potential in xenograft NOD.CB17 mice

**DOI:** 10.1186/s12885-022-09829-1

**Published:** 2022-07-11

**Authors:** Claudia Felici, Francesco Mannavola, Luigia Stefania Stucci, Loren Duda, Paola Cafforio, Camillo Porta, Marco Tucci

**Affiliations:** 1 Department of Interdisciplinary Medicine, University of Bari ‘Aldo Moro’, Bari, Italy; 2 Centre for Omics Sciences, IRCCS San Raffaele Hospital, Milan, Italy; 3 Medical Oncology Unit, Azienda Ospedaliero Universitaria Policlinico di Bari, Bari, Italy; 4 Department of Clinical and Experimental Medicine, Pathology Unit, University of Foggia, Foggia, Italy; 5 Department of Biomedical Sciences and Clinical Oncology, University of Bari ‘Aldo Moro’, Bari, Italy

**Keywords:** Melanoma, Circulating tumor cells, Phenotypic plasticity, Stemness, Metastases; NOD.CB17 mice

## Abstract

**Background:**

Innovative therapies have improved the overall survival in melanoma, although a high number of patients still experience disease progression or recurrence. Ex-vivo culture of circulating tumour cells (CTCs) represents a valuable laboratory resource for in-depth characterization of rare cell populations responsible for disease progression.

**Methods:**

CTCs from patients with metastatic melanoma were in-vitro established. Their stemness was demonstrated by both phenotypic and genotypic assays, as well as by functional studies. Xenograft experiments in NOD.CB17 mice injected with CTCs from a single patient were completed. Data were analysed by Student’s test and results expressed as mean ± SEM.

**Results:**

CTCs share the mutational profile with primary cells, an intermediate epithelial-mesenchymal transition (EMT) phenotype and high expression of the immunosuppressive factors. A subclonal CTC population exhibited stem cell properties as high aldehyde dehydrogenase 1 activity, melanosphere-forming ability, and expression of major stemness transcription factors. Xenograft experiments confirmed the CTC ability to generate melanoma in-vivo and revealed enhanced metastatic propensity.

**Conclusions:**

CTCs play a relevant role in melanoma and may actively contribute to drive the disease progression and metastasis. Thus, they are a unique potential tool for pharmacogenomic studies to guide treatment strategies in advanced disease.

**Supplementary Information:**

The online version contains supplementary material available at 10.1186/s12885-022-09829-1.

## Introduction

The most recent knowledge in genome sequencing highlighted that cancer cells accumulate genetic aberrations and epigenetic modifications favoring either the intra-tumoral or spatial heterogeneity and the inter-tumoral or temporal heterogeneity [1]. The intra-tumoral heterogeneity (ITH) mostly restrains the response to treatments and concurs to the acquired drug-resistance as result of the development of aberrant clones, thus leading to molecular, antigenic and phenotypic variability of cancer cells [2].

Malignant cells from cutaneous melanoma (CM) are highly aggressive and acquire many somatic mutations that enhance their metastatic potential. In addition, the biopsy of primary tissue from patients with CM poorly reflects the ITH and, therefore, mutational status is under certain circumstances discordant with metastasis [3]. Nowadays, circulating tumor cells (CTCs) are considered a unique tool for tracing the clonal evolution of cancer cells exposed to environmental pressure [4]. In this context, a peculiar phenotypic profile has been recently suggested for the isolation of CTCs in CM and their number apparently correlates with worse prognosis [5]. Other studies have also demonstrated that CTCs are critical for the metastatic fate of malignant cells and their seeding to distant tissues [6, 7]. However, CTC capture is still difficult as consequence of their high susceptibility to undergo to apoptosis and to suffer of immune suppression during their migration toward the pre-metastatic niche [8].

Functional assays are required to detect CTCs and explore their biological features, but results are strongly limited by their extremely low number. Further studies have been developed for the in-vitro establishment and propagation of CTCs by using short-term or long-term cultures [9–12] and a peculiar gene signature associated to outcome has been demonstrated in pre-clinical studies in breast cancer [7, 11].

Immunotherapy and BRAF inhibitors are breakthrough for the treatment of CM, but many patients develop resistance due to extensive and molecular intra- and inter-tumoral heterogeneity of melanoma cells [13, 14]. Also, patient-derived xenografts (PDX) provided relevant information for understanding this event as well as other mechanisms implicated in the resistance to targeted agents [15, 16]. However, recent data demonstrated a high discordance of gene mutations among CTCs, primary melanoma and metastasis, thus suggesting that the PDX model does not reflect the temporal heterogeneity of the disease [17].

In order to investigate the CTC heterogeneity and their biological behave, we established long-term CTC cultures from metastatic CM patients and explored the in-vitro and in-vivo properties in NOD-SCID mice, including their metastatic potential.

## Methods

### Patients and cells

Fifteen patients with metastatic melanoma (median age: 61 years, range 50-75) were enrolled before starting treatment. The study was approved by the ethics Committee of Bari (Identification code ID: 5834) and a written informed consent was obtained from each participant. Table 1 displays demographic data at time of diagnosis and enrolment. Peripheral blood (PB) from each patient was collected for both CTC detection and ex-vivo culture. The Sk-Mel-28 (ATCC HTB-72) cell line was used as control and maintained in RPMI 1640 Complete Medium (31966 Life Technologies) supplemented with 10% FBS and 1% penicillin–streptomycin.

**Table 1:** Demographic characteristics of 15 patients with metastatic melanoma

Patient ID	Sex	Primary site	Stage (diagnosis)	Breslow (mm)	Ulceration	Mitosis	BLS/CLND	Stage (enrolment)	BRAF Status	Metastastic Sites
1	M	Trunk	IIIA	0.9	N	> 1	N1a	IV – M1a(0)	WT	Sc
2	M	Arm	IIIB	1.9	Y	> 1	N2a	IV – M1b(0)	V600E	N; L; Sc
3	F	Arm	IIIB	1.2	N	> 1	N2b	IV – M1a(0)	WT	N
4	F	Arm	IIIB	1.5	Y	< 1	N2b	IV – M1c(0)	V600E	Li; Pe; Ad; Mu
5	M	Trunk	IV	1.6	Y	> 1	ND	IV – M1b(1)	V600E	L
6	M	Trunk	IIIC	11	Y	> 1	N2c	IV – M1c(1)	V6003	L; N; Bo; Li; Br
7	F	Leg	IIIB	2.1	N	> 1	N1a	IV – M1b(0)	V600E	N; L; Sc
8	M	H/N	IV	4.8	Y	> 1	ND	IV – M1c(0)	V600E	L; Li; Pe; Mu; Sc
9	M	H/N	IIIC	5.4	Y	> 1	N1b	IV – M1d(−)	V600E	Br; L; Ad; Sc; N
10	M	Trunk	IV	2.4	Y	> 1	ND	IV – M1c(−)	V600E	N; Bo; Sc; Mu
11	F	Leg	IIIB	3.6	N	< 1	N2a	IV – M1c(0)	V600E	N; Li; Bo; Sc
12	F	Trunk	IIIC	1.6	Y	> 1	N3c	IV – M1c(1)	WT	L; N; Sc; Sp
13	F	U	IV	–	–	–	ND	IV – M1d(0)	V600E	L; Br; Sc; Sp
14	M	Leg	IV	7	Y	> 1	ND	IV – M1c(0)	V600E	N; L; Li; Pe
15	M	Leg	IIIC	5.1	Y	> 1	N1a	IV – M1c(−)	V600E	N; Sc; Br; Bo; O

*Ad* Adrenal gland, *Bo* Bone, *Br* Brain, *Li* Liver, *L* Lung, *Mu* Muscle, *N* Extra-regional nodes, *O* Ovary, Pe Peritoneum, *Sc* Subcutaneous, *Sp* Spleen, *H/N* Head and Neck, *ND* Not Done, *U* Unknown, *LDH* Low:(0), *LDH high*:(1), LDH not available at diagnosis:(−)

### CTC enumeration

Melanoma CTCs were counted by DEPArray system as previously described [5]. Briefly, ten ml of peripheral blood were collected in the CellSave tube and processed using Ficoll gradient (Histopaque®-1077-Sigma-Aldrich) while CD45^+^cells and Glycophorin^+^cells were depleted by AutoMACS® cell separator (Miltenyi Biotec). The negative fraction was stained with a cocktail of three different monoclonal antibodies (MoAbs) toward putative cancer stem cell (CSC) antigens as anti-CD271 (Bio-Legend), anti-RANK (Novus Biological) and anti-ABCB5 (Novus Biologicals) MoAbs labelled with fluorescein isothiocyanate (FITC), as well as mesenchymal (M) antigens including anti-MCAM/CD146 (Becton-Dickinson), anti-N-cadherin (N-Cad; Invitrogen) and anti-CD44 (Bio-Legend) Moabs labelled with phycoerythrin (PE). Both mononuclear cells and endothelial cells were excluded from the cocktail of allophycocyanin (APC) conjugated MoAbs (anti-CD45, −CD31 and -CD34; Invitrogen). Hoechst 33342 (Sigma-Aldrich) was used to stain nuclei and exclude dead or apoptotic cells from the analysis. Finally, the sample resuspended in RPMI complete medium (10% FBS, 2 mM L-Glutamine, 1% Pen/Strep) was analysed by the DEPArray dielectrophoretic system (Menarini-Silicon Biosystems) after loading the sample into dedicated cartridges according to the manufacturer’s instructions. The cell-loaded cartridge was scanned under an automated fluorescence microscope to generate an image gallery from the Cell Browser software that allowed cells to be identified with specific fluorescence patterns and cell morphology.

### Ex-vivo culture of circulating tumor cells

To establish the CTC cultures, a parallel sample of PB from each patient was processed by both Ficoll gradient separation and negative immunomagnetic enrichment, as described above. Purified CTCs were incubated under normoxic conditions (5% CO2) in 48-well plates at 37 °C in Dulbecco’s modified Eagle’s medium/F12 (Gibco) containing insulin (20 ng/mL) (Gibco), 1% N2 complement (Gibco), epithelial growth factor (EGF: 20 ng/mL) (Gibco), 1% penicillin–streptomycin solution (Gibco), fibroblast growth factor-2 (FGF2: 10 ng/mL, Gibco) and 10% foetal bovine serum (FBS) (Gibco). The cultured cells were then fixed in 2% of paraformaldehyde (Sigma Aldrich), permeabilized by 0.1% Triton X-100 (Sigma Aldrich) and incubated overnight at 4 °C with a rabbit anti-Mela-A/MART1 MoAb (ab210546, Abcam) at 1:200 dilution and mouse anti-CD45 at 1:100 dilution (HI30, Novus Biologicals). Cells were then incubated at room temperature with an anti-rabbit FITC secondary MoAb (AP307F, Sigma-Aldrich), an anti-mouse Alexa Fluor 647 (A28181, Invitrogen), while TRITC-conjugated Phalloidin (P1951, Sigma Aldrich) and DAPI were used to visualize the β-actin and nucleus, respectively. Samples were analysed under a confocal laser scanning microscope (C2plus, Nikon Instr.) equipped with a dedicated software (NIS element software, Nikon Instruments).

### Genomic analysis by next-generation sequencing

Genomic DNA was isolated from both in-vitro established CTCs and paraffin-embedded primary tumour (DNeasy® blood & tissue Kit and QIAamp DNA FFPE Tissue Kit, Qiagen). The amount of DNA was quantified by Qubit® fluorometer (Life Technologies). Ten ng of DNA was used to prepare the barcoded library using the Ion AmpliSeq™ Library kit 2.0 and the Ion Xpress™ barcode adapters (Life Technologies). The library was purified with Agent court AMPure XP (Beckman Coulter) and quantified with the Ion Library Quantitation Kit (Life Technologies) on the StepOne Plus system (Applied Biosystem). The template preparation was completed with the Ion OneTouch™2 System and Ion One Touch ES. Finally, the sequencing was performed on PGM using Ion PGM™ Hi-Q™ Sequencing kit (Life Technologies) on the Ion 314 chip v2 and set of 500 flows standard. The reaction sequence was conducted with an Ion Torrent PGM™ system (Life Technologies) using an Ion AmpliSeq™ Cancer Hotspot Panel v2 (Life Technologies) covering 50 oncogenes and tumor suppressor genes.

### Phenotypic and gene expression analysis

The phenotype of viable CTCs was explored by flow cytometry using a panel of antigens (Supplementary Table 1) including melanoma-associated antigens (MAAs) as well as those implicated in epithelial-to-mesenchymal transition (EMT) and immune escape. Mean fluoresce intensity (MFI) was calculated. Total RNA was extracted (RNeasy kit, Qiagen) and reverse transcribed by IScript cDNA synthesis kit (Bio-Rad) and then amplified by Fast SYBR Green Master Mix on the StepOne Plus Real Time PCR (Life Technologies Inc.) using the primers listed in Supplementary Table 2. Data were normalized with respect to glyceraldehyde-3-phosphate dehydrogenase (GAPDH) to calculate 2^-ΔCT^.

### Aldehyde dehydrogenase (ALDH) activity assay

The ALDH enzymatic activity of CTCs was investigated by the Aldefluor assay kit (Stemcell Technologies) according to the manufacturer. Briefly, CTC-AT and Sk-Mel-28 cells were dissociated with trypsin and resuspended in Aldefluor assay buffer at a density of 2 × 10^6^ cells/mL. The cell suspension was treated with 5 μL/mL bodipyaminoacetaldehyde (BAAA) and incubated in a 37 °C water bath for 60 minutes. The control sample, namely ALDH-negative cells, was obtained from a 500 μL aliquot of BAAA-treated cell suspension incubated with a 15–30 μM of the ALDH inhibitor, diethylamino benzaldehyde (DEAB), and incubated as mentioned above. The ALDH1A1-positive population was identified and gated using DEAB-treated cells as our control sample. The relative increase in FITC signal of the ALDH-positive cells was determined by BD Accuri™ C6 Plus flow cytometer and analysed in three independent experiments.

### In vitro sphere formation assay

CTCs originated from patients ID:6 (CTC-AT) was seeded in ultra-low attachment 24-well plate (Corning, 3473) at a density of 10^2^ cells/mL in serum-free DMEM/F12 supplemented 1% penicillin–streptomycin solution, 20 ng/mL insulin, 1% N2 complement, 20 ng/ml EGF, 10 ng/mL FGF2. The number of spheres formed after 14 days of culture was counted using a light microscope. To assess self-renewal, spheres were dissociated and cultured in the same condition for additional two generations. The sphere formation efficiency (SFE, %) was calculated as (number of colonies counted/number of cells seeded) × 100.

### NOD.CB17 model of metastatic melanoma

To investigate the tumorigenic potential of CTCs in-vivo, we generated transduced bioluminescent cells in Red-Luc^+^-CTC-AT and Red-Luc^+^-Sk-Mel-28 by using RediFect™ lentiviral particles containing red-shifted firefly luciferase (Luc) transgene (Perkin Elmer), as previously described [18]. Luciferase expression by transduced cells was evaluated in IVIS Lumina SIII (Perkin Elmer) after supplementation of cultures with d-luciferin potassium salt (Perkin Elmer). A total of 5 × 10^5^ Red-Luc^+^ cells in 100 ml of PBS were subcutaneously transplanted into 8-week-old female NOD.CB17-Prkdcscid/J mice (*n =* 5 mice per group) purchased from Charles River (Milan, Italy). The tumour engraftment was evaluated by IVIS Lumina SIII after 3 days from inoculation and followed up-to 4 weeks. Regions of interest encompassing the signal area were defined using Living Image software and the total photons per second (photons/s) were recorded. All animal studies were carried out according to the Italian National Institute of Health guidelines on the care and use of animals for experimental procedures (Identification code ID: 29/2019-PR). The study was completed in compliance with the ARRIVE guidelines. Primary tissue and metastasis were biopsied and stored in 4% PFA (Sigma-Aldrich) for the subsequent histological analyses. Tissue biopsies of melanoma-bearing mice were cut into 4 μm sections and stained with Hematoxylin-Eosin (H&E). For immunohistochemistry, the deparaffinized sections were incubated with the following primary antibodies: anti-Ki67 (clone MIB1, Dako), anti-S100 (clone 15E2/E2, BioGenex), anti-melanosome (clone HMB45, Dako) and anti-MART1 (clone A103, Dako). Subsequently, the sections were incubated with biotinylated secondary antibodies and peroxidase-labeled streptavidin. The staining was completed with the incubation of the chromogensubstrate 3-3′-diaminobenzidine (DAB, Vector Laboratories). The images were captured on a Leica microscope (LEICA DM2500).

### Statistical analyses

Data were analysed by Student’s test and results expressed as mean ± SEM. *p* < 0.05 were considered significant.

## Results

### Melanoma CTCs were in vitro established

**Fig. 1:**
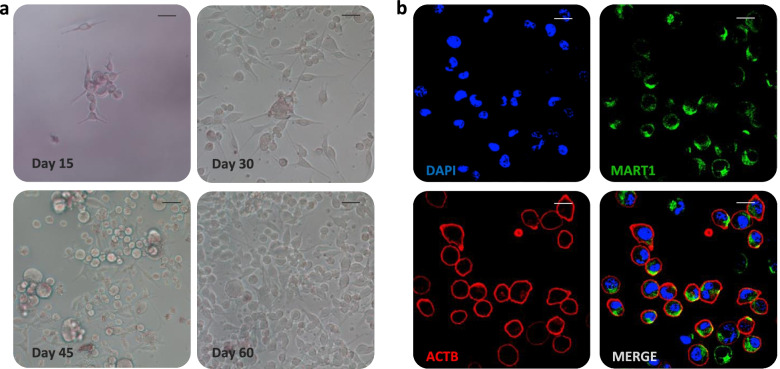
Ex vivo culture of melanoma CTCs. **A** Representative microscopic images of CTC cultures at day 15, day 30, day 45 and 60. **B** Representative panels of MART-1 (green) and beta-Actin (ACTB, red) expression by immunofluorescence staining of cultured CTCs. The DAPI (4′,6-diamidino-2-phenylindole, blue) served for counterstaining the nuclei. Magnification, 40x. Scale bar: 20 μm

Peripheral blood samples from 15 patients with metastatic melanoma, before the first-line treatment, were analyzed by the DEPArray system (Supplementary Table 3) for CTC detection. All patient samples contained variable number of CTCs with a count ≥20 cells (mean: 55.6; range, 26–102) and 9 samples (60%) showed CTC count ≥50 cells (mean, 71.8; range, 50–102). Long-term CTC culture (> 3 months) was established from one sample (patient ID:6, CTC-AT) with the highest CTC number (102 CTCs/10 mL of PB). Patient ID:6 was a 55 years-old male admitted for a rapidly enlarging, elevated, bleeding, itchy, exophytic and adjacent to pigmented lesions of 2–5 mm of larger diameter, suggestive for satellites of the trunk. The Breslow depth was 11 mm, mitosis> 1 (AJCC8th Edition) whereas endolymphatic and vessels permeation, intratumoral or peritumoral lymphocytic infiltration were undetected. The biopsy of sentinel lymph node was excluded for the rapidly growing of a clinically detectable right axillar adenopathy that was also confirmed by CT scan. Thus, he underwent to complete lymphoadenectomy. 1 month later he completed both TC and PET/CT that revealed lung and bone metastases. The mutational status revealed the BRAFV600E mutation, and thus he started first-line treatment with anti-BRAF plus anti-MEK inhibitor. He suffered clinical progression with liver and brain metastasis after 7-months of treatment. He died before receiving additional treatments for progressive liver failure. As for the in-vitro expansion of CTC-AT, cells were mostly quiescent during the first fifteen days of culture. However, in the following weeks, cells proliferated as heterogeneous colonies characterized by both fusiform adherent and suspended cells (Fig. 1 A). In order to confirm the origin of the in-vitro established cells, we performed an immunofluorescence staining for antigens specific for the melanocyte lineage as Melan-A/MART-1. A representative panel (Fig. 1 B) shows the cytoplasmic expression of Melan-A/MART-1 at variable intensity while no signal was detected for CD45, thus suggesting that cultured cells were of melanocytic origin.

Moreover, the genetic profile revealed mutation concordance between the CTCs and primary tumour with regard to the activating variant c.1799 T > A in the BRAF gene responsible for the pathogenic p.Val600Glu (V600E, rs113488022) missense mutation. In addition, CTC analysis revealed a c.215C > G transversion in *TP53* responsible for a pPro72Arg TP53 missense mutation and a c.1173A > G substitution resulting in a p.I391M missense variant in the *PIK3CA* (data not shown).

### Phenotypic plasticity of CTC-AT

**Fig. 2:**
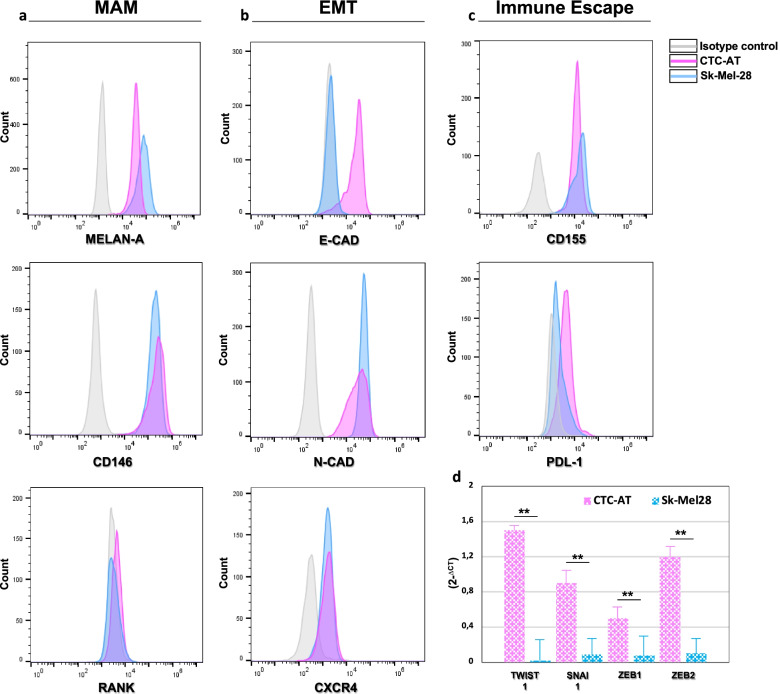
Phenotypic and molecular characterization of the CTC-AT cells. **A**-**C** The phenotypic profile of CTCs and Sk-Mel-28 was explored by flow cytometry. Representative panels show the similar expression of Melan-A (100%), CD146 (100%) and RANK (32 + 5.4%) in CTCs and Sk-Mel-28. CTCs line showed an intermediate EMT phenotype (E-CAD: 89 ± 3.9%; N-CAD: 100%) whereas Sk-Mel-28 were exclusively N-cadherin positive. The CXCR4 expression was almost similar although the MFI was lower in CTCs (MFI: 2616 vs. 3254). The CD155 and programmed cell death ligand 1 (PD-L1) expression was similar although a different MFI was revealed. **D** Real time-PCR explored the basal expression of EMT transcriptional factors in CTC-AT. Results are expressed as 2^−Δct^ and bars represent mean ± SEM. **p < 0.01. All experiments were completed in triplicate and repeated three times thus obtaining similar results

In order to define the phenotypic profile of the CTC-AT cells, the expression of different MAAs and proteins implicated in EMT and immune escape was assessed. As showed in Fig. 2 A, CTC-AT cells were characterized by variable expression of Melan-A (100%), CD146 (100%) and RANK (32 ± 5.4%). In addition, CTC-AT showed an intermediate EMT phenotype (E-CAD: 89 ± 3.9%; N-CAD: 100%) as compared to Sk-Mel-28 cells that were exclusively N-cadherin positive (100%). As shown in Fig. 2 B, CXCR4 expression was 73 ± 4.5% in CTC-AT but with a lower MFI than Sk-Mel-28 (MFI: 2616 vs. 3254). Among molecules involved in immune escape, CD155 antigen was expressed in 100% of CTC-AT (Fig. 2 C) while PD-L1 by 82 ± 2.4% of cells with a MFI of 7285 that was almost three times higher than Sk-Mel-28 (MFI: 2689). Lastly, CTC-AT cells were negative for CD45, CD34 and CD31, thus excluding their origin from circulating hematopoietic progenitors or endothelial cells (Supplementary Fig. 1).

Subsequent experiments explored the expression of transcription factors activating EMT as SNAI1, TWIST1, ZEB1 and ZEB2 by q-PCR. As expected, Sk-Mel-28 had significantly lower TF mRNA levels, confirming the mesenchymal characteristics of these cells. Conversely, q-PCR analysis revealed a different expression of TFs in CTC-AT cells. Specifically, TWIST1 and ZEB2 seem to be the factors most involved in the maintenance of the intermediate EMT phenotype of these cells (Fig. 2 D).

### CTC-AT shows hallmarks of CSCs

**Fig. 3:**
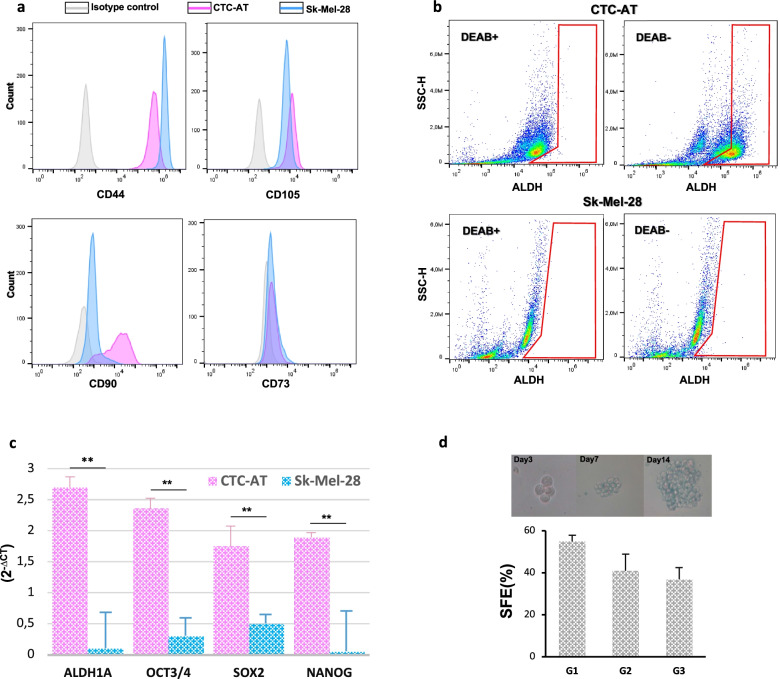
Circulating tumor cells show the molecular and functional features of cancer stem cells. **A** Typical cancer stem cell (CSC) antigens as CD44, CD105, CD90 and CD73 were highly expressed by CTC-AT in a fashion similar to Sk-Mel-28 positive control. **B** Representative panels showing ALDH1 activity in presence (+) or absence (−) of an ALDH1 inhibitor (DEAB). Cells included in the framed region are those ALDH1^+^. **C** mRNA expression levels of CSC transcription factors in CTC-AT cells and Sk-Mel-28. Results are expressed as 2^−Δct^ and bars are mean ± SEM. **p < 0.01. **D**) Melanospheres formation efficiency of the three generation (G1-G3) of CTC-AT. Data are mean ± SEM (*n* = 3). SFA: sphere formation assay. Each experiment was performed in triplicate, and representative examples are shown

Since the DEPArray analysis showed the presence of CTCs with a stem-cell phenotype, we performed a series of additional analyses to confirm the stem-like properties of CTC-AT population. Firstly, we evaluated the expression of markers specifically associated to CSCs with metastatic potential. In particular, CD44, CD105 and CD90 were highly expressed (almost 100% in all experiments), whereas CD73 was expressed in 67 ± 9% of viable population (Fig. 3 A). In parallel to these results, the measurement of ALDH1 showed that approximately half of the CTC-AT cells (43 ± 3.9%) had high enzymatic activity (Fig. 3 B). Furthermore, CTC-AT cells expressed high levels of transcription factors associated with pluripotency, such as OCT3/4, NANOG and SOX2 compared to Sk-Mel-28 (Fig. 3 C). To investigate the self-renewal ability in-vitro, we completed a SFA in anchorage-independent condition. Spheres grown gradually over time formed irregular structures, consisting of larger individual cells with a diameter ranged from 30 to 100 μm (Fig. 3 D). Moreover, we evaluated the ability of spheroid self-renewal in subsequent generations by calculating the relative SFE. As shown in Fig. 3 D, during the first generation the estimated SFE was 54.9 ± 3.1% and decreased up to 41 ± 5.1% in the second, thus remaining constant in the next generation (36.8 ± 4.1%). Overall, these results provide clear evidence that in CTC-AT cells exists a subpopulation of circulating cancer stem cells that are probably responsible of disease progression.

### CTC-AT cells showed tumorigenic potential in immunodeficient mice

**Fig. 4:**
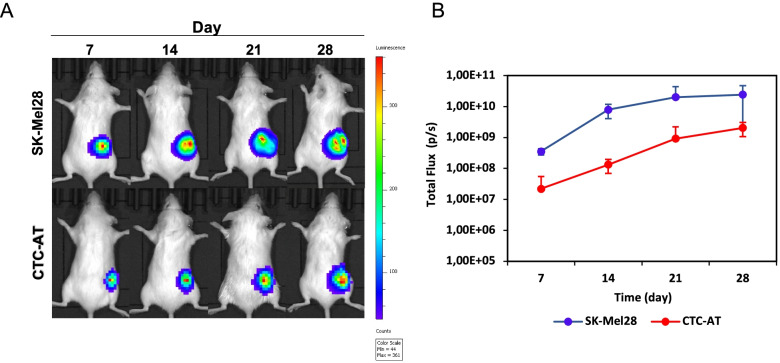
Tumor growth in xenograft model. **A** Representative bioluminescent images showing the tumor growth in xenograft NOD.CB17 mice injected with the Sk-Mel-28) and CTC- -AT up to 28 days after the inoculum. The color scale ranged from blue (just above the background noise; set to 1 × 107 photons / s / cm^2^ / sr) to red (at least 2.5 × 108 photons / s / cm^2^ / sr). **B** Dotted line represents the exponential model fit to the tumor size data points from mice. Tumor growth based on relative increase of median photon flux (photon/s) per second within ROI areas is significantly higher in mice inoculated with Sk-Mel-28 respect to CTC-AT line (*p* < 0.05)

**Fig. 5:**
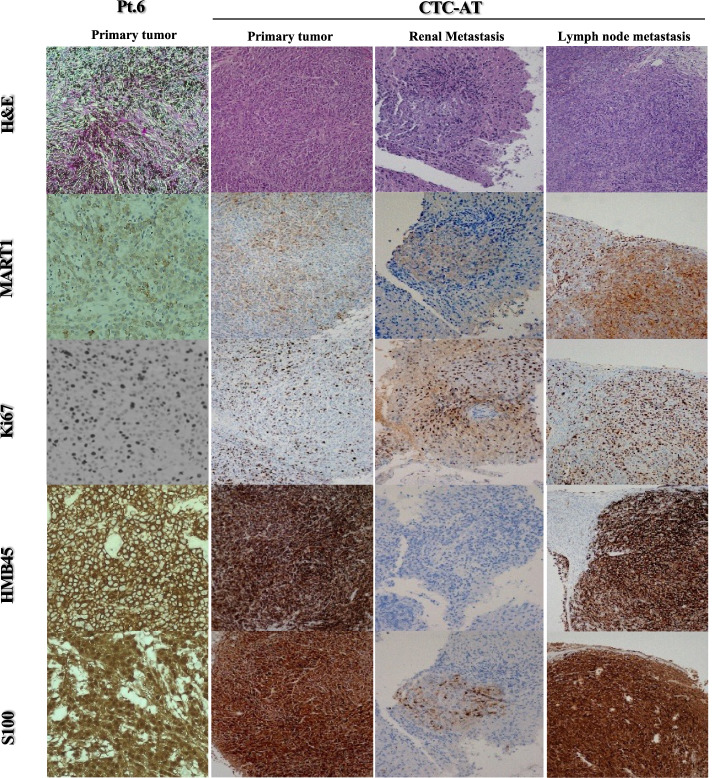
Histological analyses of primary melanoma, CTC tumor xenograft and metastasis. Representative comparative images by immune-histochemistry revealing the expression of MART1, Ki67, HMB45 and S100 by primary patient-derived melanoma (**A**), the xenograft primary tumor (**B**) and lymph node (**C**) and renal (**D**) metastasis of bearing-tumor mice. Magnification, × 40. Scale bar, 50 μm

To assess tumorigenic and metastatic potential of the CTC-AT, tumour cells were inoculated subcutaneously into immunodeficient mice. Tumour formation was revealed in all mice after the first week from injection. Tumour burden, assessed by the fold change radiance in bioluminescence imaging in-vivo, increased steadily over time until sacrifice after 28 days (Fig. 4 A and B). Presence of tumour cells in the fragments collected at the experimental endpoint was confermed by hematoxylin-eosin staining. Although the primary cells from ID:6 patient were characterized by a melanocytic proliferation with strongly pigmented spindle cells, this feature was completely lost in CTC-AT. Upon sacrificing, two out of four mice displayed nodal and renal micrometastases (Fig. 5). Primary tumour showed a strong expression of S100 and HMB45 antigens whereas levels of Ki67 and MART1 were higher in CTC-AT. On the contrary, the renal metastases were characterized by epithelioid tumor cells with an intense positivity for Ki67, intermediate positivity for MART1 and S100, while it was completely negative for HMB45. In addition, lymph node metastases retained the cell morphology of the primary tumor and were strongly positive for all markers explored (Fig. 5).

## Discussion

In recent decades, the development of new anticancer drugs based on the molecular profile of the tumour has represented a significant breakthrough in cancer management. However, many patients die from metastatic disease following the failure of treatments [19]. To date, the management of metastatic disease is hindered by limited access to cancer cells at various stages of the metastatic cascade. Recent studies suggest that ex-vivo culture of CTCs is an ideal laboratory tool for in depth characterization of subpopulations responsible for disease progression and to understand the mechanisms underlying drug-resistance [11, 20, 21] The present research provides the experimental evidence that CTCs, isolated from a patient with metastatic melanoma, give rise to a stable CTC population showing phenotypic heterogeneity and high metastatic potential.

In our study, blood samples from melanoma patients underwent immune-magnetic negative selection and CD45 negative cells were expanded in a culture medium enriched with cytokines and growth factors. Although CTCs were detected in all patients, their in-vitro stabilization was obtained only in a patient showing the the largest number of cells. Notwithstanding several groups have attempted to propagate CTCs through ex vivo cultures, most of these had short life, as 3-14 days [9–12, 20, 22–26]. The low success rate of long-term CTC cultures can be attributed to the limited number of CTCs in peripheral blood as well as technical problems related to the isolation method and culture conditions. In addition, genetic analysis of cultured cells using next-generation sequencing demonstrated that, concerning BRAF, this population harbored the primary tumour-specific molecular signatures. However, no unique genetic mutations have been identified in CTC-AT cells, as observed in other studies [26–28], while the polymorphism of of 72ArgTP53 shows a weak role in skin cancer. By contrast, a major interest concerns the PI3K pathway whose signals are pivotal for the melanomagenesis and also implicated in driving the resistance to BRAF/MEK inhibitors and immunothearapy [29, 30]. We can hypothesize that genetic analysis conducted on a limited number of genes may not provide a complete mutational snapshot. Furthermore, bulk analysis have allegedly masked the low-frequency mutations present in specific cellular subclones.

Over the past decade, several studies have shown that, during tumour spread, CTCs undergo a reversible phenotypic change, described as EMT [31, 32]. Studies of EMT effect on the behavior of CTCs have shown that the mesenchymal phenotype may facilitate the initial steps of the metastatic process but restrain their competence to become noticeable metastases [33, 34]. Therefore, it has been hypothesized that the cancer-associated EMT may not represent a complete interconversion of phenotypes but rather the achievement of an intermediate phenotype, with both epithelial and mesenchymal characteristics, that better adapts to the concept of a highly plastic state responsive to microenvironment [31]. To confirm these observations in our cell line, we studied several proteins implicated in the EMT of cancer cell. The simultaneous expression of N-cadherin and E-cadherin confirmed the intermediate EMT phenotype possessed by our population. In addition, the analysis of transcription factors associated with EMT revealed the concomitant presence of SNAI1, TWIST1 and ZEB2.

It has been proven that following the activation of the NRAS / BRAF pathway in melanoma, genes promoting cell proliferation such as SNAIL2 and ZEB2 are replaced by those regulating the migration such as ZEB1 and TWIST1. In this context, it has been recently demonstrated that the temporal and spatial cooperation of these factors influences the balance between the proliferative and invasive states of melanoma cells, thus increasing their metastatic potential [35, 36]. Although CTCs play an important role in the metastatic cascade, their survival is constantly undermined by haemodynamic forces of blood flow, death from anoikis and attacks from the immune system [8]. In addition, CTCs adopt different survival strategies including the expression of immunosuppressive molecules such as PD-L1 [37]. The PD-1 plays a pivotal role in inhibiting immune responses and promoting self-tolerance through the modulation of T-cells, favouring the apoptosis of antigen-specific T-cells and expanding regulatory T-cells. The PD-L1 is a co-inhibitory factor of the immune response that, after the binding with PD-1, restains the proliferation of PD-1 positive cells, inhibits the cytokine secretion and provokes apoptosis. In addition, PD-L1 also plays an important role in cancer as it can attenuate the host immune response to tumour cells [38]. Thus, PD-1/PD-L1 axis is responsible for cancer immune escape and makes a huge effect on cancer therapy while the PD-L1 expression by CTCs may represent a relevant issue for the early discovery of melanoma cells escaped from immune control.

Furthermore, the expression of CD155 confirmed the data of Inozume et al, revealing a different immunosuppressive mechanism triggered by the interaction between CD155 and inhibitory ITIM domains as TIGIT expressed by tumour infiltrating lymphocytes [39]. Accordingly to these results, this pathway can be included into the classical PD-1-PD-L1 co-inhibitory cascade, thus exacerbating the exhausted phenotype of T cells.

Recent studies suggested a crucial link between EMT and the acquisition of stem cell properties [40, 41]. The current hypothesis is that the metastatic potential of a tumour is due a subpopulation of CTCs, named circulating cancer stem cells (CCSC), that by an EMT intermediate phenotype, acquire stem-cell-like abilities such as self-renewal and ability to recreate the entire cell repertoire of primary tumour. Furthermore, this subset of cells was reported to be drug-resistant [40, 42] and give rise to metastatic disease [43]. The phenotype of CTC-AT showed that the majority of sub- populations were characterized by the high expression of markers as CD44, CD90, CD105 and CD7 as well as elevated ALDH activity, thus suggesting the presence of cancer stem-like cells in CTC-AT population. The hypothesis that CTC-AT cell line has CSC-like hallmarks was reinforced by the expression of stemness transcription factors such as NANOG, OCT3/4, and SOX2. Several publications report the high expression of SOX2 gene in different tumours [44, 45], thus supporting its oncogenic role. Furthermore, the expression of NANOG, OCT3/4, SOX2 by CTCs in breast cancer is directly related to disease progression [46]. The stem cell features in our line was confirmed by its ability to generate spheres in FBS-free medium. Indeed, the sphere culture was used to isolate, enrich or expand potential CSC subpopulations from cancer tissues [47, 48].

Despite the enormous difficulties to obtain stable cultures from CTCs, several studies have confirmed that they are capable of generating tumours in immunodeficient mice. In our study, we obtained tumour mass in all mice with a high growth rate and Ki67 expression. Furthermore, the discovery of lymph node and kidney micrometastases confirmed the attitude of CTCs to generate tumours in distant organs. Althoug few studies permitted to develop metastatic disease after the subcutaneous inoculation of melanoma cells [49–51], our results suggest that the CTC-AT cell line is characterized by a heterogeneous clonal populations that, showing a similar phenotype in-vitro, have a different behaves in-vivo, as recently described [52].

In conclusion, we were able to establish in-vitro a long-term culture of CTCs from a patient with metastatic melanoma. These cells showed an intermediate epithelial/mesenchymal phenotype, immune escape ability and stem cell-like characteristics. Moreover, the in-vivo study demonstrated that the CTC-AT line is tumorigenic and possesses metastasis-initiating ability. Further studies will be required to identify and characterize the subclonal populations described in our model to understand the mechanisms underlying clonal propagation and delineating mutational and gene expression profiles of organ-specific lesions.

## Conclusions

CTCs play a relevant role in melanoma progression and metastasis. These cells resemble the phenotypic and genotypic features of primary cells but acquire an intermediate EMT phenotype, immune escape ability and in most instances behave cancer stem cells. In addition, they are tumorigenic and have metastasis-initiating ability. However, further studies are required for understanding the mechanisms underlying their clonal propagation while additional pharmacogenomic assays may allow to use CTS for novel strategies for the treatment of advanced disease.

## Supplementary Information

**Additional file 1.**

**Additional file 2.**

**Additional file 3.**

**Additional file 4.**

## Data Availability

The datasets generated and/or analysed during the current study are available in the NCBI Sequence Read Archive (SRA) database (SRR19055008 and SRR19055009).
