# Supplementary material for: Circulating tumor cells from melanoma patients show phenotypic plasticity and metastatic potential in xenograft NOD.CB17 mice
Source: BMC Cancer. 2022 Jul 11;22:754. doi: 10.1186/s12885-022-09829-1 (PMC9275157; doi:10.1186/s12885-022-09829-1)
Supplement: Supplementary file 1 — Additional file 1. [file 12885_2022_9829_MOESM1_ESM.pdf]

| Target | Clone | Isotype | Label | Dilution | Manufacturer |
|---|---|---|---|---|---|
| Mart1 | MLANA/788 | Mo IgG1, κ | PE | 1:100 | Novus Biologicals |
| CD146 | P1H12 | Mo IgG1, κ | PE | 1:100 | BD Pharmingen |
| RANK | 9A725 | Mo IgG1, κ | FITC | 1:50 | Novus Biologicals |
| E-Cadherin | DECMA-1 | Rat IgG1, κ | Alexa Fluor 488 | 1:100 | eBioscience |
| N-Cadherin | 8C11 | Mo IgG1, κ | PE | 1:100 | eBioscience |
| CXCR4 | 44717 | Mo IgG2B | APC | 1:50 | R&D Systems |
| CD155 | SKII.4 | Mo IgG1, κ | APC | 1:100 | BioLegend |
| PDL-1 | 10F.9G2 | Rat IgG2b, κ | PerCPCy5.5 | 1:100 | BioLegend |
| CD44 | BJ18 | Mo IgG1, κ | PE | 1:100 | BioLegend |
| CD105 | HP6017 | Mo IgG2a, κ | PE | 1:100 | BioLegend |
| CD90 | 5E10 | Mo IgG1, κ | APC | 1:100 | eBioscience |
| CD73 | AD2 | Mo IgG1, κ | FITC | 1:50 | BD Pharmingen |
| CD45 | HI30 | Mo IgG1, κ | APC | 1:100 | eBioscience |
| CD31 | WM59 | Mo IgG1, κ | APC | 1:100 | eBioscience |
| CD34 | 4H11 | Mo IgG1, κ | APC | 1:100 | eBioscience |

**Supplementary Table 1.** Monoclonal antibodies used for flow cytometry analyses. Abbreviations: Mo:mouse; FITC: Fluorescein isothiocyanate; APC: Allophycocyanin; PE: Phycoerythrin; PerCPCy: Peridinin chlorophyll protein-cyanin.
