# Supplementary material for: Circulating tumor cells from melanoma patients show phenotypic plasticity and metastatic potential in xenograft NOD.CB17 mice
Source: BMC Cancer. 2022 Jul 11;22:754. doi: 10.1186/s12885-022-09829-1 (PMC9275157; doi:10.1186/s12885-022-09829-1)
Supplement: Supplementary file 2 — Additional file 2. [file 12885_2022_9829_MOESM2_ESM.pdf]

Gene	Forward primer	Reverse primer
GAPDH	5’-CAACGACCACTTTGTCAAGC-3’	5’-GGTGGTCCAGGGGTCTTACT-3’
TWIST1	5’-CCGGAGACCTAGATGTCATTGT-3’	5’-CCCACGCCCTGTTTCTTTGA-3’
SNAI1	5’-ACTATGCCGCGCTCTTTCCT-3’	5’-AGTCCTGTGGGGCTGATGTG-3’
ZEB1	5’-CAGGCGAGCAGTGTGACT-3’	5’-GCAAGACCGACGACCTGAT-3’
ZEB2	5’-CAGAAGCCACGATCCAGACC-3’	5’-GTCACTGCGCTGAGGTACT-3
ALDH1A	5’-TGTTAGCTGATGCCGACTTG-3’	5’-TTCTTAGCCCGCTCAACACT-3’
OCT3/4	5'-AGGTGTTCAGCCAAACGACC-3’	5'-TGATCGTTTGCCCTTCTGGC-3'
SOX2	5’-CGAGTGGAAACTTTGTCGGA-3’	5’-TGTGCAGCGCTCGCAG-3’
NANOG	5’-ATGCCTCACACGGAGACTGT-3’	5’-AAGTGGGTTGTTTGCCTTTG-3’

Supplementary Table 2: Primer sequences used for qRT-PCR
