# Supplementary material for: Circulating tumor cells from melanoma patients show phenotypic plasticity and metastatic potential in xenograft NOD.CB17 mice
Source: BMC Cancer. 2022 Jul 11;22:754. doi: 10.1186/s12885-022-09829-1 (PMC9275157; doi:10.1186/s12885-022-09829-1)
Supplement: Supplementary file 3 — Additional file 3. [file 12885_2022_9829_MOESM3_ESM.pdf]

Patient ID N=15	CTC detection by DEPArray system N=15	CSC^+	M^+	CSC^-/M^-	Ex vivo CTC culture N=1
1	85	3	47	35	
2	40	4	22	14	
3	20	0	7	13	
4	69	9	20	40	
5	29	0	29	0	
6	102	39	43	20	Yes
7	50	5	32	13	
8	44	0	13	31	
9	77	8	40	29	
10	48	2	16	30	
11	59	9	33	17	
12	52	12	15	25	
13	26	0	6	20	
14	83	9	26	48	
15	31	2	18	11	

Supplementary Table 3. CTCs isolation by DEPArray system and *ex vivo* expansion
