# Supplementary material for: Circulating tumor cells from melanoma patients show phenotypic plasticity and metastatic potential in xenograft NOD.CB17 mice
Source: BMC Cancer. 2022 Jul 11;22:754. doi: 10.1186/s12885-022-09829-1 (PMC9275157; doi:10.1186/s12885-022-09829-1)
Supplement: Supplementary file 4 — Additional file 4. [file 12885_2022_9829_MOESM4_ESM.pdf]

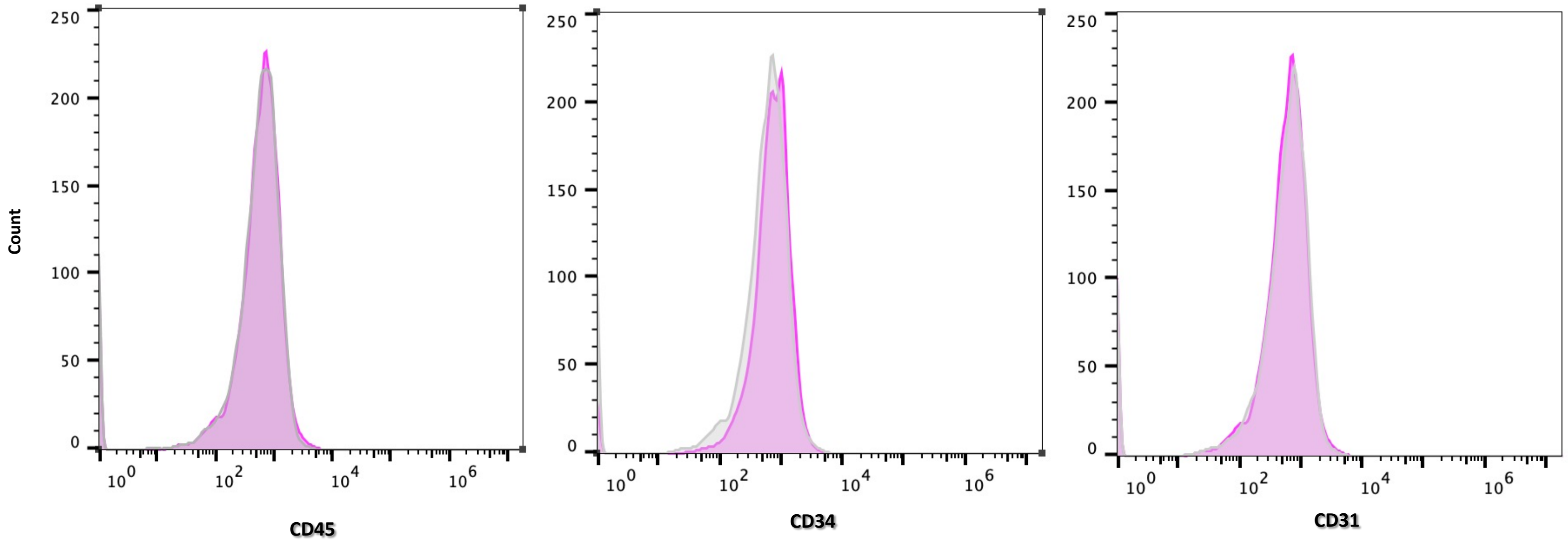

Supplementary Figure 1. Expression of hematopoietic markers by CTCs. The expression of specific hematopoietic-lineage markers as CD45, CD34 and CD31 were explored by flow-cytometry in CTCs and found negative, thus avoiding potential contaminations.
